# The Diets of Companion Cats in Aotearoa New Zealand: Identification of Obesity Risk Factors

**DOI:** 10.3390/ani11102881

**Published:** 2021-10-02

**Authors:** Rachel Forrest, Leena Awawdeh, Fiona Esam, Maria Pearson, Natalie Waran

**Affiliations:** 1 Eastern Institute of Technology, Hawke’s Bay, 501 Gloucester Street, Taradale, Napier 4112, New Zealand; mpearson@eit.ac.nz (M.P.); nwaran@eit.ac.nz (N.W.); 2 Companion Animals New Zealand, Wellington 6141, New Zealand; fiona@companionanimals.nz

**Keywords:** body condition, cats, companion animal, diet, feline, food, New Zealand, obesity, pet

## Abstract

**Simple Summary:**

Currently, there is a paucity of studies exploring the behaviours of people living in Aotearoa New Zealand regarding their responsibilities as pet owners and the factors that may influence these behaviours. In order to improve pet management and, in turn, animal welfare, we need to understand the factors that contribute to why humans behave the way they do towards their animals so that we can implement effective behaviour change programmes to benefit animal welfare. The current study aims to explore what companion cats in Aotearoa New Zealand are being fed and identify possible owner-related risk factors for developing obesity in cats. Identifying these risk factors may assist in developing future research and new approaches to obesity prevention in cats that focus on positive behaviour changes in their owners.

**Abstract:**

One in four New Zealand cats are overweight or obese, conditions associated with poor health outcomes. As part of an online survey that was conducted from January 2019 to March 2019, NZ residents aged ≥18 years were asked demographic questions along with questions related to the body condition, breed and diet of their cat/s. From the responses, possible owner-related risk factors for developing obesity were identified. Of the respondents, 65.5% (*n* = 1537) owned cat/s; the owners being more likely to be female, live rurally, or live with children. Most of the respondents fed their cat/s biscuits from the supermarket (63%) and wet food (57%). Almost half (45%) fed their cat/s specialised food from a pet shop or veterinary clinic and gave them treats, with 31% of respondents feeding their cat/s raw meat. Feeding cats a variety of food types may make it difficult to estimate the appropriate amount of each needed to avoid excess caloric intake. In addition, approximately 30% of the respondents did not agree with the correct body condition statement, revealing a need for owner education. These findings highlight important areas of cat nutrition requiring future research to better inform the development of healthy weight interventions for NZ cats.

## 1. Introduction

Globally, Aotearoa New Zealand (NZ) has one of the highest rates of pet ownership, with 41% of the population having at least one cat [1]. Despite the large cat population in NZ, there is a paucity of studies exploring the attitudes of New Zealanders towards their responsibilities as pet owners and the cultural and regionally-specific factors that may influence these attitudes. Recently, the NZ Companion Animal Trust (NZCAT) funded a Furry Whānau (family) Wellbeing Research Study [2] to explore the attitudes of pet owners in NZ towards the health and wellbeing of dogs and cats along with the factors that influence these attitudes. Here, those aspects of the study relating to the diet of cats are presented.

The domestic cat belongs to the *Felis catus* species, a small Carnivora mammal [3,4]. Domesticated cats have unique nutritional dietary requirements based on their evolutionary adaptations to a diet mainly comprised of prey [4]. Some studies have reported a positive association between the overall health of domesticated cats and the quality and type of nutrition provided [5,6]. Therefore, the type and quality of cat diet may influence cat welfare and overall general health, disease status, and even their longevity [7]. Cat obesity has been associated with many health conditions, including osteoarthritis, diabetes, and some types of cancer [8]. In NZ, it is estimated that one in four cats is overweight or obese [9].

Obesity results from excess energy consumption relative to expenditure. As animals are generally entirely reliant upon their owner for their nutrition, the ability of that human to understand when their animal is becoming overweight/obese and take appropriate action is critical. Numerous studies have reported that cat owners under- rather than overestimate their cat’s body condition score, and therefore, cannot recognise that their cat is overweight/obese [10]. Body condition scoring (BCS) is a widely used, practical method that has been validated in cats to classify animals according to their level of body fat [11]. Variability in reporting an accurate BCS has been reported between cat owners and veterinary professionals, with studies suggesting that cat owners regularly underestimate BCS [10,12,13].

An increase in domestic cat obesity has been reported worldwide [10,14,15], which is highly concerning for cat welfare. Historically, in 1970, 6.5–12% of cats were reported to be obese and/or overweight [16]. Two decades later, the prevalence of overweight and/or obesity has increased to 18.9–52% in domestic cats in the USA, England, Canada and Australia [13,17,18,19]. A similar pattern of cats being overweight/obese has been reported in NZ [13]. In 1993, 26% of domestic cats were reported to be overweight and/or obese in the North Island compared to 63% in 2007 [13,20]. Although some of the studies recognised a similar obesity pattern in human populations, the exact association was not characterised [14,21]. Although there are a large number of studies regarding the prevalence and risk factors associated with human obesity, studies addressing this in cats are scarcer.

According to Companion Animal New Zealand (CANZ) (2020), cats are the most popular companion animal in NZ, with 41% of households sharing their home with at least one cat and 1.219 million cats in households across the country. Cat ownership was found to be highest among females (43%), people living outside of Wellington and Auckland in the North Island (48%) and in rural areas generally across NZ (52%), households with adults between 45 and 54 years of age (53%), children at home (48%), a higher income bracket (46%), and that are NZ European (46%). Māori households were less likely to have a cat/s (36%). The current study aims to explore what companion cats in NZ are being fed and identify possible owner-related risk factors for the development of obesity in cats. This information will help inform interventions aimed at maintaining a healthy weight in pet cats.

## 2. Materials and Methods

### 2.1. Participants and Data Collection

An online survey was developed and offered to the people of NZ via Survey Monkey©. The 2019 NZ Pet Survey was developed by Eastern Institute of Technology (EIT) researchers Dr Rachel Forrest (Associate Professor, Research) and Maria Pearson (Lecturer and Researcher, School of Nursing) in consultation with Natalie Waran (Professor—One Welfare, EIT), EIT Centre of Veterinary Nursing staff, Patu^TM^ Aotearoa staff, Jael Reiri (Kaitiaki Māori, Lecturer, School of Nursing, EIT) and Mark Farnworth (Associate Professor—Animal Welfare, Nottingham Trent University). The Survey Monkey© link was distributed through email networks and distributed online via social media (in particular Facebook) and Patu^TM^ websites (which have a high Māori engagement), with a snowball sampling approach being used to promote the survey and recruit participants [22]. The survey remained open for approximately three months (from 8 January 2019 to 31 March 2019).

The survey asked the following demographic and cat diet- and body condition-specific questions:

- Are you: Female, Male, Gender diverse?
- To which ethnic group do you belong? Tick as many as apply: NZ European, Other European, Māori, Samoan, Cook Island Māori, Tongan, Niuean, Chinese, Indian, Don’t know, Other (please specify)
- To which age range do you belong? 18–24 years, 25–34 years, 35–44 years, 45–54 years, 55–64 years, 65–74 years, 75–84 years, 85 years and over
- In which region do you currently live? Northland, Auckland, Bay of Plenty, Waikato, Taranaki, Gisborne, Hawke’s Bay, Manawatu-Wanganui, Wellington, Nelson, Marlborough, Tasman, West Coast, Canterbury, Otago, Southland, Other (please specify)
- In the last 12 months, what was your total household income? Less than $14,000, $14,001–48,000, $48,001–70,000, $70,001–100,000, Over $100,000, Would rather not say
- What is your highest completed qualification? National Certificate level 1, National Certificate level 2, National Certificate level 3, National Certificate level 4, Trade certificate, Diploma or Certificate level 5, Advanced trade certificate, Diploma or Certificate level 6, Teachers Certificate or Diploma, Nursing Diploma, Bachelor Degree, Bachelor (Hons), Postgraduate Certificate/Diploma, Master’s Degree, PhD, Other (please specify)
- Including yourself, how many adults (over 18 years of age) live at your usual address? 0, 1, 2, 3, Other (please specify)
- How many children under 18 years of age live at your usual address? 0, 1, 2, 3, 4, 5, Other (please specify)
- Were you born in New Zealand? Yes, No
- Where were you brought up? Tick as many as apply: In a town or city, On a farm or rurally, On a lifestyle block, Other (please specify)
- Where do you live now? In a town or city, On a farm or rurally, On a lifestyle block, Other (please specify)
- Do you own a cat? Yes, No
- How many cats do you own? 1, 2, 3, 4, Other (please specify)
- My cat is a pedigree. (Selected if it applied)
- What do you feed your cat/s? Tick all that apply: My cat is fed cat biscuits from the supermarket, my cat is fed specialised cat food from a pet shop or vet clinic, My cat is fed raw meat, My cat is fed wet food, My cat is fed table scraps/human food, My cat is fed food that I have cooked for them, My cat is fed treats, Other (please specify)
- Health/Care section. Please choose the option (Strongly agree, Agree, Neutral, Disagree, strongly disagree) that most closely describes how you feel about the following statements. Cats should: have a specialised diet from a pet shop or vet clinic; have ribs, hips, and a spine that are not visible but are easily felt; Please provide further explanation if required.

With regard to the food type/s fed, in NZ, the phrase cat biscuits refers to dry food or kibble (as opposed to a dry treat) and wet food is typically bought in cans or foil packets and is available as pate or in chunks with jelly, gravy or broth.

### 2.2. Statistical Analysis

Respondents were not required to answer all the questions and were provided with the option to skip questions as they desired. For the demographic questions, where a respondent chose “Other, please specify” if possible, the information was recoded into the appropriate category. For example, those who did not select European but identified as British were recoded as European. Various categories were collapsed as necessary for statistical analyses to maintain adequate sample sizes.

The qualitative data from the diet-specific questions were analysed independently by at least two researchers for emergent themes and categories using a general inductive approach [23]. The quantitative data in the form of forced responses to survey questions were analysed using descriptive statistics. Where appropriate, inferential statistics such as correlations (Spearman’s Chi-square), z-tests, and binary logistic regressions were employed to explore the associations between the demographic data and the participant responses to the questions asked. All statistical analyses were carried out using IBM SPSS Statistics (version 25) software.

Forward stepwise binary regressions were used to explore if gender (female, male), ethnicity (Māori, NZ European, Other), age (18–24 years, 25–34 years, 35–44 years, 45–54 years, 55–64 years, 65–74 years, 75–84 years), income range (<$14,000, $14,001–48,000, $48,001–70,000, $70,001–100,000, >$100,000), qualification level (1–10), being brought up rurally (yes, no), currently living in a town (yes, no), the number of children, and or the number of adults present in the household impacted on cat ownership or were associated with an owner’s choice of diet for their cat/s.

For each of the Health/Care statements, cross-tabulations along with Chi-square and z-tests (α = 0.05) were used to explore if the respondent answer choices were associated with gender, ethnicity, age range, household income bracket, qualification level, whether or not they had a child or children, whether or not they had a rural upbringing, or whether they were currently living in a town/city.

Any owner-related risk factors that were identified were compared to any demographic factors that were found to impact on cat ownership.

## 3. Results

### 3.1. Demographic Description of the Respondents

Nationally, 2744 people responded to the online survey. A description of the demographics of these respondents can be found in the *Furry whānau wellbeing: Working with local communities for positive pet welfare outcomes* report prepared for the NZCAT [2]. In short, 92.3% of the respondents were female, and the majority of the respondents identified as NZ European (83.4%), with 8.3% (*n* = 229) of respondents identifying as Māori. Māori are the indigenous people of NZ. All age ranges were represented by both males and females, and by Māori and non-Māori, with a decline in respondent numbers for the older age ranges. New Zealand is divided into 16 regions for local government purposes; however, poor representation of males in some regions meant that this factor could not include in any of the inferential statistical analyses. Of the respondents that disclosed their household income (*n* = 2251), there was a relatively even distribution across the income brackets above 14 K, and there were similar percentages of Māori and NZ European, and male and female respondents, in each. Likewise, there were similar percentages of respondents for each of NZ Qualification Authority (NZQA) levels for Māori and NZ European, and male and female respondents, in each. Of the respondents, 25% indicated that they had lived rurally as a child (had a rural upbringing) and at the time of the survey, 76% of the respondents lived in a town or city. Of those respondents that had a rural upbringing, 68% currently dwelled in a town or city. No differences in the percentage of respondents who had a rural upbringing or town/city dwelling due to ethnicity or gender were observed. How these demographics impacted on the likelihood of owning cat/s is presented in the next section.

### 3.2. Cat Companionship

A total of 2358 respondents answered the pet companionship questions. Of these, 37.5% (*n* = 885) respondents owned both dog/s and cat/s and 28% (*n* = 652) owned cat/s only. Nine per cent of the respondents indicated that their cat was a pedigree, with the likelihood of owning a purebred cat increasing with qualification level (odds ratio 1.120, *p* = 0.012). Of the respondents, the information from 1800 could be used in the binary regression analysis for cat ownership (yes, no). Gender (odds ratio 0.587, *p* = 0.003, with males being less likely to own a cat) and the number of children (odds ratio 1.276, *p* < 0.001) were retained in the model. As the number of children increased, there was an increased likelihood of having cat companion(s). Not surprisingly, place of residence was associated with the number of household cats, with those living in a town or city owning fewer pets (*p* < 0.029). For cats, household income level was associated with the number of household cats (*p* = 0.003) along with the age range (*p* = 0.024). Bonferroni corrected pairwise comparisons revealed that those with an income under 14 K had more household cats, with those in this income bracket having an average of three cats compared to the two-cat average for each household of the other income brackets. Those respondents in the 18–24 years, 75–84 years and 85 plus years of age brackets had an average of one household cat compared to the two-cat average for each of the other age ranges.

### 3.3. Demographics of Cat Owner Respondents

Of the survey respondents, 1537 owned a cat/s, 9.4% indicating that their feline companion/s were pedigree. A total of 1533 cat-owning respondents disclosed their gender and age group. Of these, 93.8% were female, 5.5% male and 0.2% gender diverse; 23.5% selected 45–54 years, 21.9% selected 35–44 years, 18.3% selected 25–34 years, 15.9% selected 55–64 years, 12.5% selected 18–25 years and 7.8% selected an age group above 64 years. Of those cat-owning respondents that disclosed their ethnicity (*n* = 1514), 82.2% were NZ European and 8.1% were Māori. Household income brackets were indicated by 1278 respondents, with 5.3% indicating less than 14 K, 20.7% indicating between 14 and 48 K, 20.8% indicating between 48 and 70 K, 25.0% indicating between 70 and 100 K, and 28.2% indicating over 100 K. The number of adults per household was provided by 1525 cat-owning respondents, with 19.3% of households having one adult, 54.9% having two adults, 15.4% having three and the remainder having four or more adults. The number of children per household was provided by 1528 cat-owning respondents, with 69.3% not having any children, 13.9% having one child, 12.6% having two, the remainder having three or more children. One-quarter (25.2%) of the cat-owning respondents (*n* = 1534) were brought up rurally. At the time of the survey, three-quarters (74.8%) of those cat-owning respondents (*n* = 1532) lived in a town or city.

### 3.4. Feeding Practices

Table 1 displays the percentage of respondents who selected each diet choice and, based on the binary logistic regression analyses, which variables were indicated to be associated with these choices. A majority of the respondents selected biscuits from the supermarket (63%) and wet food (57%). Almost half of the respondents (45%) also fed their cat/s specialised food from a pet shop or veterinary clinic and gave them treats. A respondent’s cat/s were more likely to be fed biscuits from the supermarket as age range and or the number of children in the household increased and less likely as qualification level increased. The opposite was true for the likelihood that a respondent’s cat was fed specialised cat food from a pet shop or veterinary clinic. Gender, age-range and qualification level influenced if a respondent selected that they fed their cat/s raw meat, with females (32% versus 22% of males) and those with increased age being more likely to feed their cat raw meat and a decreased likelihood as qualification level increased. As the number of adults and children in the household increased, so did the likelihood that the respondent selected ‘My cat is fed table scraps/human food’. This likelihood decreased with increased household income. With increased household income, a decreased likelihood that ‘My cat is fed food that I have cooked for them’ was selected. Whereas as the age range increased, there was an increased likelihood of a cat having a home-cooked meal. As the age range and number of children increased, there was a decreased likelihood that ‘My cat is fed treats’ was selected, but an increased likelihood if the respondent was a town/city dweller (46% versus 40%).

Of the 1525 respondents that answered the questions regarding what they feed their cat/s, 84 provided further comment. Table 2 shows the themes that emerged from these comments for each food choice with representative quotes. Several respondents pointed out that good quality and specialised diet cat biscuits could be purchased from the supermarket. Some of the respondents shared that they had tried food purchased from a pet shop or veterinary clinic but that it either did not suit their cat/s or was too expensive. Several comments also highlighted that supermarket cat biscuits were used in combination with other food types. This theme also emerged in the comments about specialised cat food, raw meat, and wet food. Raw meat appeared to be occasionally fed and often associated with the respondent’s own dinner routine. Many of the respondents commented that they feed their cat/s fish and that their cat/s were hunters and ate whatever prey they caught. Several respondents highlighted the fact their cat was a thief. Health reasons were provided as an explanation for providing home-cooked pet food. Treats ranged from being used as a reward to reinforce desired behaviours to overt spoiling. Wet food was also indicated to be a treat in some households. Other themes that emerged from comments highlighted that some owners had cats on different diets due to their specific needs or they did not feed their cat/s any specific type of diet. Two respondents noted that their cats also ate dog food.

### 3.5. Attitudes towards Appropriate Body Condition and Specialised Pet Food

The respondents were asked to indicate their level of agreement with best practice statements describing appropriate body conditions and another about the need for a specialised diet. The results are shown in Table 3. The majority of the respondents either strongly agreed or agreed that cats (70.2%) should have ribs, hips, and a spine that are not visible. In contrast, those who strongly agreed or agreed with the statement that cats should have a specialised diet from a pet shop or veterinary clinic were in the minority (Table 3). Several significant associations were detected (Chi-square *p* < 0.001). For cats, associations were observed regarding “should have a specialised diet” with a lower percentage of those respondents with children either strongly agreeing (6% vs. 9%) or agreeing (13% vs. 19%) and a higher percentage disagreeing (21% vs. 11%) when compared to the percentage of respondents who do not have children. Again, the age range also influenced the level of agreement with a higher percentage for 18–24 and 25–34 year age group respondents agreeing, and a lower disagreeing and strongly disagree than the 35–44 and 45–54 year age groups. Additionally, a lower percentage of those in the 18–24 and 25–34 age range strongly disagreed compared with the 55–64 and 65–74 age ranges.

## 4. Discussion

This study interrogated the relevant data from a recent national survey funded by the NZ Companion Animals Trust [2] and identified two possible risk factors that may contribute to companion cats in NZ becoming overweight or obese, namely, incorrect owner perception of body condition and feeding a varied diet making control of caloric intake challenging. These factors are discussed in detail below. As is typical of online surveys, the majority of respondents were female [24,25]. However, previous studies in NZ have found that “No matter what the companion animal, it is typically the female household head that takes responsibility for pet food buying and feeding duties”, p. 14 [26]. Thus, the gender bias in this sample was appropriate for this investigation. Nevertheless, caution must be taken when interpreting the statistical analyses where gender is identified as a significant factor, with the results highlighting areas of further research. There was not a representative sample of Māori in the survey respondents, with 8.3% being well below the 16.7% of the national population estimate in 2020 [27]. While the findings of this study will help to inform future research areas and the development of a healthy weight intervention for cats in NZ, research with an indigenous perspective is paramount.

The current study identified incorrect owner perception of body condition as a potential risk factor for cats becoming overweight or obese. The BCS is a validated scale that can classify cats into three groups: under-weight (1–3), ideal-weight (4–5) and overweight and obese (6–9) based on visual observation and palpation of superficial boney prominences [11,28]. A score of 5 is associated with the ability to palpate the ribs with minimum fat coverage with there being a strong correlation between BCS and body fat mass [11]. Many studies have reported that owners underestimating feline BCS can be considered a risk factor for cats being overweight/obese [10,12,18,29,30,31]. In the current study, a total of 70.2% of respondents correctly indicated that cats should have ribs, hips, and a spine that are not visible but are easily felt. However, one in ten respondents disagreed with this, identifying incorrect owner perception of appropriate body condition as a potential risk factor for cats becoming overweight or obese. In addition, a further two out of ten respondents choose a neutral response (neither agreeing or disagreeing with the correct body condition statement), indicating a lack of confidence in their knowledge around this subject. Collectively, these findings suggest the need for educational programmes for NZ cat owners that enable them to correctly identify feline BCS and detect any changes regarding their pets BCS, allowing the owner to modify their cat’s diet. To accompany any educational programme, pre- and post-intervention studies should be undertaken to determine the level of agreeance between the BCS for a cat by the owner and a veterinary professional, evaluate the effectiveness of the intervention and also estimate the percentage of cats in NZ that are overweight/obese.

Sixty-two per cent and 57% of cat owners stated in the online survey that they buy dry (biscuits) and wet (canned) foods from the supermarket. Additionally, 45% indicated that they bought specialised food from a pet shop or veterinary clinic and while the food type was not disclosed a walk into any pet store or veterinary clinic in NZ will reveal that the bulk of the pet food they sell is dry. The preference for feeding dry food aligns with previous studies which found that the popularity of dry over wet food could be due to affordability, convenience and long shelf life [18,32]. The current study did not investigate the rationale behind feeding the selected cat food but we would speculate that perceived food quality also influenced diet.

Numerous studies have reported a positive association between cat obesity and the predominant consumption of dry pet food as is, regardless of the quality of the food, and it has been suggested this is a consequence of dry food typically having higher calories per gram in comparison to wet food [18,29,32,33,34]. However, none of these studies [18,29,32,33,34] reported the quantity of dry food that the cats consumed; therefore, the reported cat obesity may also have been a consequence of more food being consumed to reach satiety due to gastric extension. This phenomenon has been studied in dogs but not cats [35,36]. Some studies have also found an association between the type of food and the feeding frequency in cats [37,38]. Given that both the quality and quantity of food ingested influence energy intake, the association between feeding protocols factors (type of food, frequency and quantity provided) must be considered when evaluating the effect of diet on body weight and condition. No data were collected regarding the feeding frequency, duration, and exact amount the cat was fed in the current study and these dietary aspects should be the focus of future research in the area of cat nutrition in NZ.

Raw meats were reported to be fed to cats by 31% of respondents in the current study, with raw meat often being referred to as being occasional and or a treat. Wales et al. (2019) have reported that any particular type of raw feeding decreased the risk of obesity and provided beneficial enrichment [39]. The latter was potentially due to the extra time needed to chew the raw materials. Furthermore, other studies have supported Wall et al. (2019) and claimed raw meats have nutritional and behavioural benefits compared with conventional processed diets [39,40,41]. In contrast, another study has found that there is a positive association between obesity risk and the frequency of feeding raw meat and fish [19]. In addition, raw meat could act as a vector for some potential pathogens for pets and their owners [40]. In the current study, males were less likely to feed their cat/s raw meat and treats. Taken together, as part of a healthy weight programme for NZ cats, owners, in particular males, could be encouraged to occasionally provide their cat/s raw meat and fish from trusted sources to ensure it is free of pathogens as part of their feline diet.

In this study, respondents also indicated that typically wet food was viewed as a treat and fed in combination with dry food unless health reasons dictated otherwise. Previous studies have found that cats prefer wet (canned) over dry food due to moisture similarity with meat [42,43], thus being consistent with use as a treat. However, Rowe et al. (2015) have suggested that the regular daily consumption of wet foods may reduce obesity risk. This could be due to the high water content combined with low calorific concentration found in cats’ wet food meaning that cats need to consume a larger quantity of wet cat food to exceed their daily required energy to gain weight [44,45]. While avoiding dry food and providing wet food may decrease obesity risk in cats, this strategy may negatively impact oral health conditions and increase the risk of periodontal disease [46,47,48]. Some studies found that providing hard chewable materials helped control plaque and periodontal disease [46,48]. Additionally, other studies proposed a positive association between wet diets and periodontal disease [49,50]. In the 2019 NZ Pet Survey, only 44% of the respondents indicated that their cat’s teeth are cleaned by the veterinarian when needed and 9% with their own cat’s teeth implying that for approximately half the respondent’s, dry food plays an important role in the oral health of their cat/s.

Collectively, findings presented in this discussion, support the provision of both dry, wet and raw food. Interestingly, as age increased in the present study, so did the likelihood of the respondent’s cat/s being fed biscuits from a supermarket, raw meat, and homecooked food suggesting the older the respondent the more likely their cat/s were being provided with a varied diet. A varied diet can often make it difficult to control the caloric intake; therefore, consideration regarding the quality and quantity of both and ensuring that cats do not consume more than their daily requirement. That stated this is challenging if a cat supplements its diet by hunting prey. Cat access to the outdoors and the impact of their predatory behaviours on wildlife is a contemporary issue in NZ [51,52,53]. While several studies have suggested that unrestricted outdoor access improves cats’ overall health and welfare [10,12,44] and that limited outdoor access can be considered a potential risk factor for obesity in cats [19,44], this needs to be balanced against “the potential negative impacts of cats on communities, other species, and ecosystem” p5 [54]. In the 2019 NZ Pet Survey, 86% and 55% of respondents indicated their cat/s had free indoor-outdoor access during the day and at night, respectively [2]. Furthermore, 6% of the respondents indicated that they confined their cat/s at daytime while 27% confined their cat/s inside at night [2].Those who kept their cats inside did so to keep their cats safe and or protect birdlife, and some mentioned that their cat/s had access to a conservatory or catio [2]. Future studies to investigate the effect of activity level and enrichment provided for the cats on the risk of obesity are needed. In addition, the correlation between the feeding protocols and the access to outdoor activity needs to be explored.

Ohland et al. (2018) found that obese cats were more likely found in two-adult households [29]. Interestingly in this study, the number of adults in a household was not found to be associated with food type (dry, wet, raw), where the food was bought from (supermarket, pet shop or veterinary clinic) or whether a cat/s is feed treats, home-cooked meals or table scraps. Thus, the number of adults in a household is unlikely to be an obesity risk factor in NZ. Conversely, the number of children in a house was found to be positively associated with both the likelihood of owning cat/s and the likelihood of cat/s being fed biscuits from the supermarket and table scraps and negatively associated with the likelihood of cat/s being fed a specialised diet from a pet shop or veterinary clinic or treats. These results could reflect the time constraints due to caring for both children and animals, making convenience a main driver in feline diet-related choices. This notion is further supported by the fact that no associations were detected with income and food type (dry, wet, raw), where the food was bought from (supermarket, pet shop or veterinary clinic) or whether a cat/s is feed treats. Wall et al. (2015) suggested that owners with jobs, thus higher income, might select a more expensive diet to provide better quality food but found no evidence between obese cats and the owner’s income [12]. In this study, the only association with income and feline diet was that, with increasing income, cat/s were less likely to be fed table scraps or food specifically cooked for them.

Qualification level, on the other hand, may influence the quality of diet provided to cat/s, with increasing qualification level being associated with a decreased likelihood of being fed cat biscuits from a supermarket or wet food and an increased likelihood of being fed specialised cat food from a pet shop or veterinary clinic. Interestingly, with increasing qualification level, there was also an increased likelihood the respondent owned a pedigree cat. Pure breed cats have been associated with low obesity risk among cats [12]. This could be because breeders and breed societies often provide extensive educational resources for owners and continued support and advice throughout the animal’s lifetime.

The difference in the sources where cat owners obtained their cat food was one of the interesting findings of the current study. Overall, 45% reported that they feed their cat food from specialised pet shops or veterinary clinics, independent of income bracket. The usage of pet stores and veterinary clinics as a source of information for diet selection is in agreement with previous studies [6,55]. While the reasons for buying from a pet store or veterinary clinic were not investigated, this finding suggests that these sites can be used in the promotion and or provision of healthy weight interventions to all socioeconomic groups.

## 5. Conclusions

This study has identified two possible owner-related risk factors that may contribute to companion cats in NZ becoming overweight or obese: incorrect owner perception of body condition and feeding a varied diet making control of caloric intake challenging. The identification of these risk factors highlights important areas of cat nutrition requiring future research to better inform the development of healthy weight interventions for NZ companion cats. Further research that explores BCS, its perception by owners versus veterinary professions, and its associations with activity, and type and frequency, quality and quantity of food are required, along with studies from an indigenous perspective. Guidelines for providing cats with a varied diet for optimal weight, nutritional value, dental health and environmental enrichment also need to be developed as most of the cat owners involved in this study provided their cat/s with a combination of food types and treats. Regardless of socioeconomic group, the findings of this study suggest that for approximately half of the cat owners in NZ, veterinary clinic and pet store staff are well placed to play an essential role in encouraging them to monitor their pet’s BCS and ensure their cat is fed appropriate amounts of a high-quality diet, including treats for environmental enrichment. However, for those households with children, which are also more likely to have cat/s, a healthy weight intervention for cats needs to be conveniently accessed, for example at a supermarket, as cat owners from these households are less likely to buy food from a pet store or veterinary clinic.

## Figures and Tables

**Table 1:** Positive responses to the question “Which of these apply to your cat/s?” and factors and or variables that impact on the likelihood (odds ratio) of a positive response.

What Do You Feed Your Cat/s?	Number *n*	Percentage %	Associated Variables and Odds Ratio * (*p*-Value)
My cat is fed cat biscuits from the supermarket	945	62%	Age range 1.164 (<0.001) Qualification level 0.941 (0.026) Number of children 1.330 (<0.001)
My cat is fed specialised cat food from a pet shop or vet clinic	682	45%	Age range 0.893 (0.008) Qualification level 1.102 (<0.001) Number of children 0.756 (<0.001)
My cat is fed raw meat	480	31%	Gender: male/female 0.455 (0.021) Age range 1.204 (<0.001)
My cat is fed wet food	871	57%	Age range 1.207 (<0.001) Qualification level 0.920 (0.002)
My cat is fed table scraps/human food	199	13%	Household income 0.807 (0.002) Number of adults 1.242 (0.016) Number of children 1.244 (0.009)
My cat is fed food that I have cooked for them	78	5%	Age range 1.253 (0.025) Household income 0.745 (0.012)
My cat is fed treats	683	45%	Gender: male/female 0.398 (0.003) Age range 0.882 (0.003) Number of children 0.704 (<0.001) Town/city dwelling 1.389 (0.024)
My cat is fed cat biscuits from the supermarket	945	62%	Age range 1.164 (<0.001) Qualification level 0.941 (0.026) Number of children 1.330 (<0.001)
My cat is fed specialised cat food from a pet shop or vet clinic	682	45%	Age range 0.893 (0.008) Qualification level 1.102 (<0.001) Number of children 0.756 (<0.001)
My cat is fed raw meat	480	31%	Gender: male/female 0.455 (0.021) Age range 1.204 (<0.001)
My cat is fed wet food	871	57%	Age range 1.207 (<0.001) Qualification level 0.920 (0.002)
My cat is fed table scraps/human food	199	13%	Household income 0.807 (0.002) Number of adults 1.242 (0.016) Number of children 1.244 (0.009)
My cat is fed food that I have cooked for them	78	5%	Age range 1.253 (0.025) Household income 0.745 (0.012)
My cat is fed treats	683	45%	Gender: male/female 0.398 (0.003) Age range 0.882 (0.003) Number of children 0.704 (<0.001) Town/city dwelling 1.389 (0.024)

* An odds ratio of greater than 1 indicates an increased likelihood of a positive response, whereas an odds ratio of less than one indicates a decreased likelihood of a positive response.

**Table 2:** Thematic analysis of comments provided about respondents’ cat diets in the 2019 New Zealand Pet Survey.

Category		Representative Quote/s
Supermarket biscuits	Good quality biscuits	“I don’t feed them the cheap biscuits from the supermarket. They get Purina”
	Specialised diet available	“Special urinary diet from the supermarket”
	In combination with other food types	“Biscuits for In-door cats from Countdown and RC Dental biscuits from work (Vet clinic)”
	Only viable option	“… the only food, that I’ve found, which doesn’t flare up his allergies is from the supermarket”
Specialised cat food	Health reasons	“Our three cats are each prescribed a different medicated food for health issues by our vet”
	Online/imported	“Blackhawk, brought in from Australia.”
	Expensive	“Black hawk was too rich for her and senior pet store foods $$$”
	In combination with other food types	“Mix of vet/supermarket dry food”
Raw meat	Varied meat types	“Tuna and chicken” “Rabbits and fish” “Fresh fish” “… add chicken livers & lambs heart/kidney …”
	Prey	“My cat is a hunter and often supplements his diet” “They also eat whatever they hunt (mostly rabbits)”
	During dinner preparation	“Occasional treats inc raw snapper and raw beef if we are cooking some”
	Occasional: As a treat, fussy	“The raw meat is very occasional, he is a bit fussy about different food”
	In combination with other food types	“Mainly raw meat, some commercial wet food, few biscuits and treats each day (they are spoilt)”
Wet food	As a treat/Occassinal	“Wet food as a treat once or twice a week”
	Health reasons	“My cat was in an accident and can only eat wet food”
	In combination with other food types	“Dry food from specialist. Wet food from supermarket”
Table scraps/ human food	Occasional	“She gets occasional bits of meat or fish off my dinner plate”
	Stolen	“One cat steals human food off the plate so have to monitor her around food”
Home-cooked pet food	Recommended	“When recommended by the vet… I cooked softer foods that she could eat more easily”
	Supplement	“I often cook chicken in the crockpot for them as the broth is very good for them”
Treats	As a reward/for training	“Treats only for when clipping claws or giving medication”
	Occasional: meat, dairy	“Occasionally licks of yoghurt, ice cream”
	Part of the dental routine	“Treats are aimed to help with cleaning teeth”
	Unhealthy	“My cats very rarely get treats, as they aren’t healthy”
	Spoilt	“treats each day (they are spoilt)”
Other	Dog food	“She also likes eating dog biccies that are always available” “Dog roll if she chooses and milk”
	Variety	“My cat has variety in food.”
	Individualised diets	“One cat is fed dry biscuits and the other wet food (neutered male prone to cystitis)”

**Table 3:** Percentage of 2019 New Zealand Pet Survey respondents are selecting each level of agreement for the statements regarding appropriate body conditions and specialised pet food.

Statement—Cats Should:	N	Strongly Agree	Agree	Total Agree	Neutral	Disagree	Strongly Disagree	Total Disagree
have ribs, hips, and a spine that are not visible but are easily felt	2250	27.1%	43.1%	70.2%	20.2%	6.6%	3.0%	9.6%
have a specialised diet from a pet shop or vet clinic	2247	8.0%	17.2%	25.2%	58.1%	13.3%	3.3%	16.7%

## Data Availability

The data presented in this study are available on request from the corresponding author.
